# FIGO good practice recommendations on modifiable causes of iatrogenic preterm birth

**DOI:** 10.1002/ijgo.13857

**Published:** 2021-09-14

**Authors:** Catalina M. Valencia, Ben W. Mol, Bo Jacobsson, the FIGO Working Group for Preterm Birth, Joe Leigh Simpson, Jane Norman, William Grobman, Ana Bianchi, Stephen Munjanja, Andrew Shennan

**Affiliations:** ^1^ Department of Obstetrics and Gynaecology Universidad CES Medellín Colombia; ^2^ Fundared‐Materna Bogotá Colombia; ^3^ Department of Obstetrics and Gynaecology Monash University Clayton Australia; ^4^ Aberdeen Centre for Women's Health Research Institute of Applied Health Sciences School of Medicine Aberdeen UK; ^5^ Department of Obstetrics and Gynecology Institute of Clinical Science Sahlgrenska Academy University of Gothenburg Gothenburg Sweden; ^6^ Department of Obstetrics and Gynecology Sahlgrenska University Hospital Gothenburg Sweden; ^7^ Department of Genetics and Bioinformatics Domain of Health Data and Digitalization Institute of Public Health Oslo Norway

**Keywords:** elective delivery, iatrogenic preterm birth, modifiable causes

## Abstract

Iatrogenic preterm birth is a planned delivery that occurs before 37 weeks of gestation due to maternal and/or fetal causes. However, in some cases, such deliveries also occur with no apparent medical indication. The increasing numbers of iatrogenic preterm deliveries worldwide have led researchers to identify modifiable causes that allow the formulation of preventive strategies that could impact the overall preterm birth rate. The present document contains the FIGO (International Federation of Gynecology and Obstetrics) Working Group for Preterm Birth recommendations, aiming to reduce the rates of iatrogenic preterm birth based on four of the most common clinical scenarios and issues related to iatrogenic preterm delivery. The working group supports efforts to identify the contribution of iatrogenic preterm delivery to the overall preterm birth rate and encourages health authorities to establish preventive measures accordingly. We encourage care providers to maintain single embryo transfer policies to prevent multiple pregnancies as a substantial contributor of iatrogenic preterm birth. The working group also recommends that efforts to reduce unnecessary cesarean sections must be warranted, and mechanisms to ensure the appropriate time of delivery and strengthening of education and communication processes must be pursued.

## Synopsis

Iatrogenic preterm birth contributes substantially to preterm birth rates globally and can be mitigated by identifying modifiable factors and formulating and supporting preventive strategies.

## 1 INTRODUCTION

Iatrogenic preterm delivery, also called provider‐initiated preterm birth, is defined as a birth that occurs before 37 weeks of gestation due to a planned delivery (induction of labor or cesarean section in the absence of spontaneous labor or rupture of membranes). According to reports, iatrogenic preterm delivery constitutes approximately 30%–35% of all preterm deliveries and may vary according to the region.^1^, ^2^, ^3^, ^4^ In the past decades, the rates of iatrogenic preterm deliveries have been increasing. As a result, it has become the leading cause of preterm delivery in some countries, reaching almost 50% of all preterm births.^2^, ^3^, ^4^, ^5^

The causes of iatrogenic delivery vary according to the region of the world, but in general they can be divided into four main groups:

- obstetric complications (e.g. hypertensive disorders of pregnancy, placental conditions, antepartum hemorrhage)
- fetal causes (e.g. fetal distress, fetal growth restriction, structural malformations)
- maternal medical conditions (e.g. heart disease, nephropathy, cancer, sepsis)
- non‐medically indicated iatrogenic preterm delivery.^2^, ^5^

The incidence of iatrogenic preterm delivery is increasing worldwide. Some of the factors that may influence this phenomenon include the increase in maternal age, which is associated with more significant comorbidities and obstetric complications; the increase in the prevalence of obesity; the use of assisted reproductive techniques with the consequent rise in multiple pregnancies, which also carries an increased risk of obstetric complications in singleton pregnancies, including an increased rate of cesarean delivery—a risk factor for subsequent complications such as placenta previa and placenta accreta.^2^, ^6^ In addition, doctors’ behavior also plays a role, as some obstetricians underestimate the risks of preterm delivery.

In some countries and regions, the main contributors of iatrogenic delivery have been identified and reported. For example, in China, causes such as hypertensive disorders of pregnancy, placenta previa, and multiple pregnancy are the most frequent, whereas in Brazil, hypertensive disorders of pregnancy, placental abruption, and diabetes play a significant role in the number of iatrogenic preterm births.^7^, ^8^ Based on these data, strategies have been proposed to address potentially modifiable risk factors for iatrogenic preterm delivery in order to reduce iatrogenic preterm delivery.^8^, ^9^

## 2 CLINICAL SCENARIOS AND ISSUES

### 2.1 Iatrogenic preterm delivery for maternal, medical, and obstetric complications

Some pre‐existing maternal conditions and obstetric complications may require delivery before 37 weeks of gestation to ensure the safety of the mother and/or the baby. However, the evidence supporting recommendations for the timing of delivery for most of these conditions is limited and primarily based on expert consensus. Therefore, this decision‐making process often requires individualization. The prevalence of the different causes of iatrogenic preterm delivery varies depending on world region.^1^, ^4^ However, some of the most common maternal medical conditions and obstetric complications that may require indicated preterm birth are:

- hypertensive disorders of pregnancy
- placental and umbilical cord anomalies.

Preventing the conditions mentioned above is an ongoing challenge. Strategies such as reducing cesarean delivery rates would probably have an impact on the incidence of placenta previa or accreta; policies to reduce obesity in women would decrease the rates of gestational diabetes; and appropriate screening and use of low‐dose aspirin in selected populations has been proven to reduce the prevalence of pre‐eclampsia.^10^ However, there are reasons to believe that doctors’ attitudes and clinical behavior are the most critical factors.

*Recommendation: Efforts should be directed to identifying the contribution of iatrogenic preterm delivery to the overall rate of preterm delivery and its causes in each country. We encourage health authorities to establish action plans, screening programs, evidence‐based preventive measures, and health policies to target modifiable risk factors to prevent iatrogenic preterm delivery*.

### 2.2 Iatrogenic preterm delivery for fetal causes

Fetal development is a complex process that involves the interaction of genetic and environmental factors. Alterations at any step along the way can lead to fetal complications that may require early delivery to improve the chances of a healthy child. Fetal conditions such as fetal distress and fetal growth restriction secondary to impaired placental function and monochorionic multiple pregnancies are among the most common fetal causes of iatrogenic preterm delivery.

Preventing fetal causes of preterm delivery requires further research. However, assisted reproductive technologies have led to an increase in multiple pregnancies and, therefore, a related increase in preterm birth rates. Singleton pregnancies conceived using assisted reproductive technologies are also at increased risk of pregnancy complications. According to the Human Multiple Births Database, the global twin rate increased by a third (9.1 to 12.0/1000 deliveries) between 1980–1985 and 2010–2015.^11^ The clinical impact of the increase of multiple pregnancies in terms of preterm birth, as reported by the Centers for Disease Control and Prevention (CDC), is that three of every five twin babies are born preterm (six times the rate for singletons) and one of every four preterm twins is admitted to the neonatal intensive care unit (five times the rate for singletons). Therefore, optimizing assisted reproductive technologies is a mandatory step toward reducing iatrogenic prematurity, particularly the adoption of single embryo transfer.

*Recommendation: Continue and strengthen policies such as single embryo transfer to regulate assisted reproductive technologies worldwide, and promote and support research to understand and prevent fetal causes of preterm birth*.

### 2.3 Recommendation for the timing of iatrogenic delivery for common pregnancy conditions

While in each pregnancy the mother and fetus require individualized care, a general rule can be defined for common pregnancy conditions. These rules are based on large randomized clinical trials conducted in recent years comparing induction of labor and expectant management. As is apparent from Table 1, iatrogenic delivery is not required for any of the common pregnancy conditions and appropriate monitoring is advised instead, perhaps with the exception of pre‐eclampsia, in which delivery between 34 and 37 weeks can be considered,^12^, ^13^ while for women with chronic hypertension the recommendation based on non‐randomized data is 38 weeks.^14^ In pregnancies complicated by growth restriction at term, the DIGITAT study showed that the optimal timing of induction is around 38 weeks,^15^ while in pregnancies with early‐onset growth restriction without fetal distress, earlier induction does not improve outcomes.^16^ Similarly, for pregnancies complicated by macrosomia, induction of labor at 38 weeks improved outcomes compared to expectant management.^17^ While RCTs are lacking for studies complicated by diabetes, it can be assumed that in the presence of macrosomia, induction of labor at 38 weeks improves outcomes.

**TABLE 1:** Indicative gestational age of delivery for different pregnancy complications

Condition	Gestational age recommended for planned delivery	Evidence from literature
Pregnancy‐induced hypertension	39 weeks	HYPITAT I and II^12^
Pre‐eclampsia	34–37 weeks	HYPITAT I and II^12^ Phoenix^13^
Chronic hypertension	38 weeks	Population‐based study^14^
Fetal growth restriction without fetal distress	38 weeks	DIGITAT,^15^ GRIT^16^
Large baby (including diabetes)	38 weeks	DAME^17^
Preterm Prelabour Rupture of Membranes (PPROM) (without GBS)	37 weeks	PROMPT, PROMEXIL I & II^18^
Uncomplicated dichorionic twin pregnancy	37 weeks	Individual participant data meta‐analysis^19^
Uncomplicated monochorionic twin pregnancy	37 weeks and 0 days	Individual participant data meta‐analysis^19^
Uncomplicated singleton pregnancy	41 weeks	Index and Swepis,^20^ ARRIVE^21^

For pregnancies complicated by preterm prelabour rupture of membranes (PPROM) without GBS or other signs of infection, expectant management until 37 weeks improves neonatal respiratory outcomes.^18^ Careful monitoring for signs of infection is warranted as women with PPROM between 34 + 0/7 and 36 + 6/7 weeks who undergo expectant management are more likely to have an antepartum hemorrhage or chorioamnionitis.^18^ For women with uncomplicated twin pregnancies, individual participant data meta‐analysis of cohort studies shows the optimal timing of delivery to be 37 weeks for dichorionic pregnancy and 37 weeks + 0 days for monochorionic pregnancy.^19^ Finally, in women with uncomplicated singleton pregnancies, two large RCTs definitively showed that induction of labor should be offered at 41 weeks,^20^ whereas the ARRIVE study suggests that induction of labor at 39 weeks improves outcomes.^21^

Three things should be stressed. First, and most important, these are general rules of thumb for pregnancies complicated by a condition but with otherwise non‐compromised mother and fetuses. Of course, individual findings regarding the condition of the mother or fetus justify earlier delivery. Second, apart from pre‐eclampsia, all recommended gestational ages are at or beyond 37 weeks, which should stimulate careful consideration around scheduling women for iatrogenic preterm delivery. Third, it should be considered that progression of pregnancy, in general, improves cognitive performance of the offspring.^22^

### 2.4 Previous cesarean delivery and preterm delivery

Cesarean delivery (CD) rates have increased worldwide over the past decades, particularly in middle‐ and high‐income countries. It has been reported that between 1990 and 2014 the global average CD rate increased 12.4% (from 6.7% to 19.1%), with an average annual increase of 4.4%.^23^ In the secondary analysis of the Multicountry Survey on Maternal and Newborn Health (WHOMCS), the WHO has demonstrated that previous cesarean deliveries are associated with increased risk of preterm birth and complications that lead to preterm delivery, such as uterine rupture (aOR 7.7; 95% CI 5.5–10.9), morbidly adherent placenta (aOR 2.6; 95% CI 2.0–3.4), and placenta previa (aOR 1.8; 95% CI 1.5–2.1).^23^, ^24^

The reasons for the increase in cesarean rates are multifactorial and poorly understood. However, factors that may play an essential role for some countries are health systems dynamics and limited resources, making caesarean delivery a more convenient mode of delivery, sociocultural issues like women's fear of pain or pelvic relaxation after vaginal delivery, and maternal and clinician preferences.

*Recommendation:* *To reduce preterm delivery related to previous cesarean complications, efforts should be made on a multilevel basis to avoid unnecessary cesarean sections*.

## 3 NONMEDICALLY‐INDICATED PRETERM DELIVERY

In some studies, and particularly in low‐ and middle‐income countries, there is a significant percentage of iatrogenic deliveries between 34 and 36 weeks.^4^, ^5^ However, a clear indication is not always recorded. This happens due to the absence of, or lack of adherence to, clinical practice guidelines, or practice based on personal experience rather than evidence‐based for the treatment of medical complications. It is well known that the morbidity of a late preterm infant born between 34 and 36 weeks of gestation is seven times greater than a full‐term infant.^1^, ^2^ Therefore, the decision to deliver a preterm infant should balance the risks of morbidity and perinatal mortality of prematurity against the possible maternal and fetal consequences of continuing a pregnancy. One of the strategies aiming to reduce the number of late iatrogenic preterm and early‐term births is elective induction of labor and elective cesarean section after 39 weeks of gestation.^25^ This policy has been adopted and proven successful in countries like the United States.^26^

Another cause of iatrogenic preterm delivery could be the lack of appropriate dating of pregnancy. It is well known that the first trimester ultrasound, when performed by properly trained personnel, constitutes the most accurate method to estimate gestational age. However, in the absence of a proper ultrasound examination before 22 + 0 weeks of gestation, the pregnancy is considered as suboptimally dated and therefore at greater risk for iatrogenic preterm birth.^27^

*Recommendation: Mechanisms for implementing and ensuring a first trimester ultrasound for appropriate dating of pregnancy as well as adherence to clinical practice guidelines for appropriate delivery timing in different medical, fetal, and obstetrical conditions should be considered. Strengthening the patient education and communication processes to achieve good decision‐making processes must be pursued*.

## CONFLICTS OF INTEREST

Catalina M. Valencia reports no conflicts of interest. Ben W. Mol reports an investigator grant from NHMRC; consultancy for ObsEva; and research funding from Guerbet, Ferring, and Merck KGaA. Bo Jacobbson reports research grants from Swedish Research Council, Norwegian Research Council, March of Dimes, Burroughs Wellcome Fund and the US National Institute of Health; clinical diagnostic trials on NIPT with Ariosa (completed), Natera (ongoing), Vanadis (completed) and Hologic (ongoing) with expendidures reimbused per patient; clinical probiotic studies with product provided by FukoPharma (ongoing, no funding) and BioGaia (ongoing; also provided a research grant for the specific study); collaboration in IMPACT study where Roche, Perkin Elmer and Thermo Fisher provided reagents to PLGF analyses; coordination of scientific conferences and meetings with commercial partners as such as NNFM 2015, ESPBC 2016 and a Nordic educational meeting about NIPT and preeclampsia screening. Bo Jacobbson is also Chair of the FIGO Working Group for Preterm Birth and the European Association of Perinatal Medicine's special interest group of preterm delivery; steering group member of Genomic Medicine Sweden; chairs the Genomic Medicine Sweden complex diseases group; and is Swedish representative in the Nordic Society of Precision Medicine.

## AUTHOR CONTRIBUTIONS

All authors and the FIGO Working Group for Preterm Birth drafted the concept and idea of the paper. CM wrote the first version of the manuscript. BWM and BJ revised various versions of the manuscript. All authors and working group members commented on the manuscript and approved the final version of the manuscript.

## MEMBERS OF THE FIGO WORKING GROUP FOR PRETERM BIRTH, 2018–2021

Bo Jacobsson (Chair), Joe Leigh Simpson, Jane Norman, William A. Grobman, Ana Bianchi, Stephen Munjanja, Catalina M. Valencia, Ben W. Mol, Andrew Shennan.
